# H_2_O_2_‑Responsive Boronic Ester-Modified Mesoporous Silica Nanocarrier for TfR Mediated Tumor-Specific Drug Delivery Applications

**DOI:** 10.1021/acsabm.5c00645

**Published:** 2025-06-23

**Authors:** Hsiao-Yen Lee, Natesan Thirumalaivasan, Shu-Pao Wu

**Affiliations:** † Department of Applied Chemistry, 34914 National Yang Ming Chiao Tung University, Hsinchu 30010, Taiwan, Republic of China; ‡ Department of Periodontics, Saveetha Dental College and Hospitals, Saveeta Institute of Medical and Technical Sciences (SIMATS), Chennai 600077, India

**Keywords:** targeted drug delivery, transferrin, mesoporous silica nanoparticles, hydrogen peroxide, cancer cells

## Abstract

This study introduces a drug delivery system utilizing boronic ester-functionalized mesoporous silica nanoparticles (**MSNP-BA-Tf**) for targeted cancer therapy. By conjugating transferrin (Tf) to the MSNP surface, the system actively targets cancer cells via transferrin receptor (TfR)-mediated endocytosis. The incorporation of boronic ester linkages enables hydrogen peroxide (H_2_O_2_)-responsive drug release, enhancing therapeutic efficacy. In vitro experiments demonstrated that **MSNP-BA-Tf** effectively delivered doxorubicin (DOX) to cancer cells while sparing normal cells, as confirmed by fluorescence imaging and cytotoxicity assays. In vivo studies using a colon cancer xenograft model showed that **DOX@MSNP-BA-Tf** inhibited tumor growth more effectively than free DOX. These findings highlight **MSNP-BA-Tf** as a promising nanocarrier for cancer therapy, offering targeted and controlled drug delivery through active targeting and H_2_O_2_ responsiveness.

## Graphical abstract

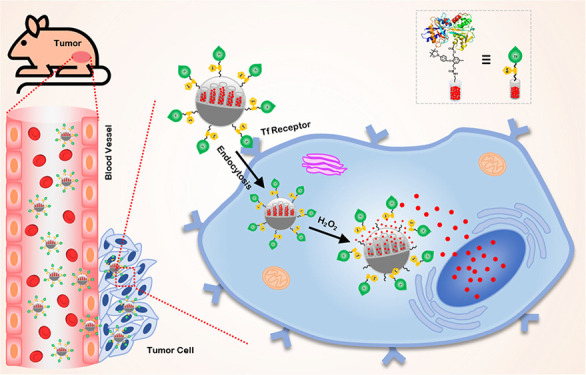

## Introduction

Cancer is a major public health concern, with cases and deaths increasing globally.^1^ Despite advancements in treatment, challenges such as metastasis and the disease’s complexity persist, highlighting the need for new therapeutic approaches. Traditional treatments like surgery and radiation are primarily effective for localized cancers. Chemotherapy, though widely used, often causes severe side effects and is limited by drug resistance. These issues have shifted focus toward targeted therapies designed to minimize adverse effects and overcome resistance mechanisms. A promising strategy involves utilizing nanoparticles, which can accumulate in tumors due to their leaky vasculature, enhancing the efficacy of treatment delivery.^2^

Advancements in nanotechnology have significantly expanded the range of nanocarriers used in drug delivery systems (DDS), enhancing the precision and efficacy of therapeutic interventions. Nanocarriers—such as polymeric nanoparticles, liposomes, mesoporous silica nanoparticles (MSNPs), and micelles—have become integral components of modern drug delivery systems.^3^ Stimuli-responsive nanoparticles are engineered to activate under specific intracellular conditions, enabling precise and controlled drug release within cancer cells. This targeted approach not only reduces toxic side effects but also enhances therapeutic efficacy against cancer cells.^4^

MSNPs are recognized for their chemical stability and biocompatibility.^5–7^ Their size and pore structure can be customized, facilitating the attachment of targeting molecules such as folic acid (FA),^8,9^ hyaluronic acid (HA),^10–13^ and transferrin (Tf).^14,15^ Transferrin is a glycoprotein that transports iron via its receptor, transferrin receptor 1 (TfR1), which is commonly overexpressed on rapidly dividing cancer cells, making transferrin an effective targeting ligand.^16^ By employing targeted drug delivery strategies, therapies can more precisely differentiate between cancerous and normal cells, thereby enhancing the efficacy and reducing the side effects of cancer treatments. Furthermore, MSNPs can be engineered with “gatekeepers” that regulate the release of their therapeutic cargo in response to various environmental stimuli, including enzymes,^17,18^ light,^19,20^ pH levels,^21–24^ redox conditions,^25^ and temperature.^26^ Reactive oxygen species (ROS), such as hydrogen peroxide (H_2_O_2_), are particularly relevant in this context due to their elevated presence in tumor cells resulting from intrinsic oxidative stress.^27^ This characteristic has driven the development of reactive oxygen species (ROS)-responsive drug delivery systems (DDS) designed to selectively release therapeutic agents within cancerous environments, thereby minimizing exposure to healthy tissues and reducing associated side effects. Incorporating reactive oxygen species (ROS)-responsive elements into drug delivery systems (DDS) enables targeted drug release, minimizing toxicity and side effects on healthy cells, and offering controlled drug release.^28–33^ These advantages make ROS-responsive DDSs a promising alternative to traditional chemotherapy agents. Their potential applications in biomedicine have garnered significant interest.

Our research focuses on developing a targeted drug delivery system using transferrin-gated, H_2_O_2_-responsive mesoporous silica nanoparticles (MSNPs), termed MSNP-BA-Tf. MSNPs are ideal nanocarriers due to their substantial pore size and high surface area, facilitating efficient loading and transport of therapeutic agents. In our approach, transferrin was conjugated to MSNPs via a boronic ester linkage, effectively preventing premature cargo release. The overexpression of transferrin receptors (TfR) on various cancer cells—including those of the liver, breast, colon, and lung—enhances the specificity of this targeting strategy. The effectiveness of the MSNP-BA-Tf system was evaluated through confocal imaging studies using DOX@MSNP-BA-Tf in HCT116 and MCF-7 cancer cell lines. These experiments validated both the specific targeting capability of transferrin and the responsiveness of MSNP-BA-Tf to H_2_O_2_ in vitro. Additionally, we assessed the biocompatibility and tumor-targeting properties of DOX@MSNP-BA-Tf, demonstrating its potential to selectively deliver drugs to cancer cells while diminishing off-target effects on normal cells. Developing targeted drug delivery systems like MSNP-BA-Tf holds promise for enhancing cancer treatment by improving drug efficacy, reducing toxicity, and maximizing therapeutic outcomes.

## Experimental Section

### Synthesis of MSNP-NH_2_

Mesoporous silica nanoparticles (MSNPs) were synthesized using a conventional sol–gel method in an alkaline environment. Initially, 900 mL of deionized water was stirred continuously at 800 rpm and heated to 80 °C. A solution containing 2 g of cetyltrimethylammonium bromide (CTAB) and 7 mL of 2 M sodium hydroxide (NaOH) in 50 mL of deionized water was prepared. Over 2 h, a mixture of 5.0 mL of tetraethyl orthosilicate (TEOS) dissolved in 43 mL of deionized water was gradually added with continuous stirring. The resulting white precipitate was centrifuged at 10,000 rpm for 10 min and washed multiple times with methanol and deionized water. To introduce amine functionality, the MSNPs were grafted with 3-aminopropyltriethoxysilane (APTES). A suspension was prepared by mixing 300 mg of MSNPs with 300 μL of APTES in methanol. This mixture was refluxed at 80 °C for 24 h under a nitrogen atmosphere. The resulting amine-modified MSNPs (MSNP-NH_2_) were obtained by centrifugation at 10,000 rpm for 10 min and washed repeatedly with methanol and deionized water. To remove CTAB, the MSNP-NH_2_ nanoparticles were suspended in methanol containing hydrochloric acid (HCl) and refluxed for 24 h. The particles were then centrifuged at 10,000 rpm for 10 min and thoroughly washed with methanol and deionized water. Finally, the nanoparticles were dried at 80 °C for 24 h.

### Synthesis of MSNP-BA

To prepare boronic acid-functionalized silica nanoparticles (MSNP-BA), a round-bottom flask was first evacuated under nitrogen with sodium carbonate (Na_2_CO_3_, 2 g, 18.86 mmol) and triphosgene (500 mg, 1.68 mmol) at 0 °C for 1 h. Subsequently, 15 mL of anhydrous dichloromethane (DCM) and 100 mg of amine-modified MSNPs (MSNP-NH_2_) were added to the flask. The mixture was stirred continuously at room temperature for 4 h. Boronic acid (BA), serving as a trigger site responsive to hydrogen peroxide (H_2_O_2_), was dissolved in anhydrous DCM and gradually added to the mixture to functionalize the silica nanoparticles with boronic acid (MSNP-BA). After 24 h of stirring, the mixture was centrifuged at 10,000 rpm for 10 min. The nanoparticles were washed with methanol and deionized water to remove residual reagents, then dried at 80 °C for 24 h before storage (Figure 1.

**Figure 1:**
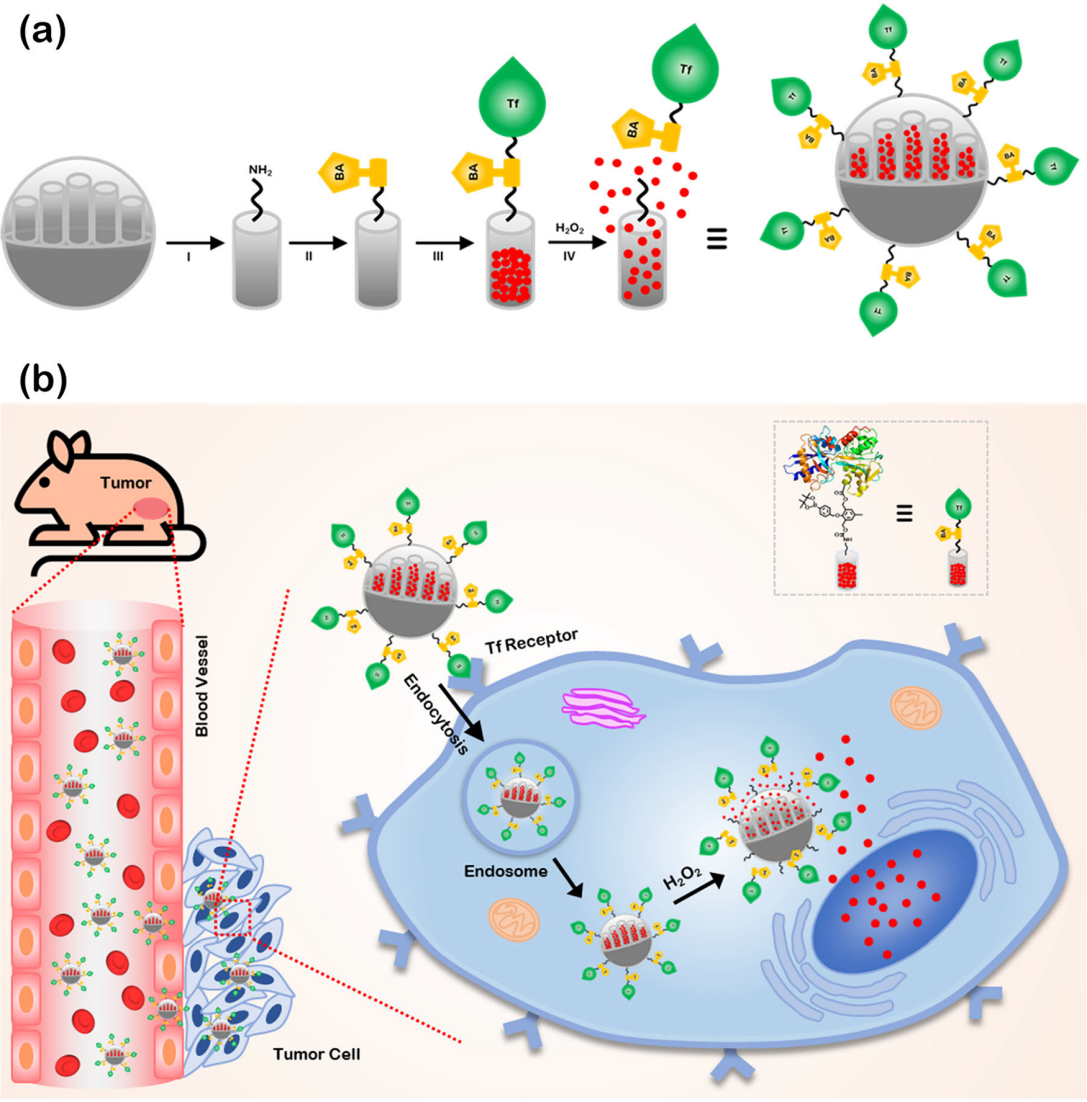
(a) Schematic of the **MSNP-BA-Tf** modification: (I) amine functionalization; (II) H_2_O_2_-responsive functionalization with a boronic ester group; (III) loading of drugs and gating with transferrins in **MSNP-BA-Tf**; (IV) drug release triggered by H_2_O_2_. (b) Mechanism illustrating how **DOX@MSNP-BA-Tf** reaches cancer cells and undergoes release facilitated by endogenous H_2_O_2_ within the cell nucleus.

### Synthesis of DOX@MSNP-BA-Tf

To prepare doxorubicin (DOX)-loaded boronic acid-functionalized mesoporous silica nanoparticles (MSNP-BA), 10 mg of MSNP-BA was suspended in 1 mL of deionized water containing either 2 mg of rhodamine B or DOX. The mixture was reacted for 24 h at 5 °C, followed by separation via centrifugation and vacuum drying. Next, a coupling solution was prepared by dissolving 10 mg of transferrin (Tf), 48 mg of *N*,*N*′-dicyclohexylcarbodiimide (DCC), and 28 mg of 4-dimethylaminopyridine (DMAP) in dry dichloromethane (DCM). This solution was stirred for 3 h at 0 °C in the dark. Subsequently, the DOX-loaded **MSNP-BA** was added, and the reaction was stirred for an additional 48 h at 5 °C in the dark. The mixture was then centrifuged and washed with deionized water to remove impurities. Finally, the DOX-loaded MSNP-BA conjugated with Tf (**MSNP-BA-Tf**) was vacuum-dried.

### Cytotoxicity Assay

The MTT assay was employed to assess the cytotoxicity of various treatments on MCF-7, HCT116, and HCK293 cells. Cells were seeded in 96-well plates and cultured for 24 h. After washing with PBS, medium containing free DOX, **DOX@MSNP-BA-Tf**, or **MSNP-BA-Tf** was added, and incubation continued for another 24 h. MTT reagent was then added, and after a 4-h incubation, the formed formazan crystals were dissolved in DMSO. Absorbance at 570 nm was measured to determine cell viability. Cell viability was calculated using the formula:

$$\mathrm{Cell}\;\mathrm{Viability}\;(\%)=[(A_{570}\;\mathrm{of}\;\mathrm{treated}\;\mathrm{cells})/(A_{570}\;\mathrm{of}\;\mathrm{the}\;\mathrm{blank})]\times 100$$

where *A*_570_ represents the absorbance at 570 nm.

### Cellular Image

MCF-7, HCT116, and HEK293 cells were cultured in Dulbecco’s Modified Eagle Medium (DMEM) or Minimal Essential Medium (MEM), each supplemented with 10% fetal bovine serum and 1% antibiotics, at 37 °C in a 5% CO_2_ atmosphere. After 24 h, cells were seeded into six-well plates and incubated with MSNP-BA-Tf for 0.5, 2, 4, or 6 h. Following incubation, the medium was removed, and cells were washed with phosphate-buffered saline (PBS) to eliminate residuals. Nuclei were stained with DAPI for 20 min.

### In Vivo Anticancer Efficacy

Female BALB/c nude mice were subcutaneously injected with approximately 1 × 10^7^ HCT116 cells to induce tumor growth. Tumor volume was calculated using the formula: *V* = *a* × *b* ^2^/2, where '*a*’ is the tumor length and '*b*’ is the tumor breadth. When tumors reached a volume of 50 mm^3^, the mice were randomized into groups and administered the following treatments: PBS (100 μL), free DOX (100 μL of 50 μg/mL), MSNP-BA-Tf (100 μL of 2.5 mg/mL), and DOX@MSNP-BA-Tf (100 μL of 2.5 mg/mL). Over a 14 day period, body weight and tumor size were measured daily. Upon study completion, tumors were excised and subjected to hematoxylin and eosin (H&E) staining for histological examination.

## Results and Discussion

The synthesis of transferrin-capped H_2_O_2_-triggered mesoporous silica nanoparticles (**MSNP-BA-Tf**) is outlined in Scheme 1. Mesoporous silica nanoparticles (MSNPs) were initially synthesized using the sol–gel technique, employing tetraethyl orthosilicate (TEOS) as the silica source and structure-directing agents such as cetyltrimethylammonium bromide (CTAB). Aminopropyl-modified MSNPs (MSNP-NH_2_) were then prepared by grafting 3-aminopropyltriethoxysilane (APTES) onto the MSNP surface. The APTES content affects the surface charge and morphology of the nanoparticles, with higher APTES ratios leading to changes in particle shape and size. Subsequently, MSNP-NH_2_ were reacted with a boronic acid (BA) compound and triphosgene to yield mesoporous silica nanoparticles functionalized with boronic acid (MSNP-BA). The BA group is linked to p-hydroxybenzyl alcohol, forming an arylboronic ester. Finally, transferrin (Tf) was conjugated to MSNP-BA via esterification using dicyclohexylcarbodiimide (DCC) and 4-dimethylaminopyridine (DMAP), resulting in transferrin-capped MSNPs (**MSNP-BA-Tf**) capable of loading therapeutic agents such as doxorubicin (DOX). Upon exposure to hydrogen peroxide (H_2_O_2_), the arylboronic ester group undergoes oxidation and subsequent hydrolysis, releasing the phenol. This process initiates a quinone methide rearrangement, leading to the degradation of the linkage and the controlled release of the loaded cargo, as depicted in Scheme S2 (in the Supporting Information).

**Scheme 1:**
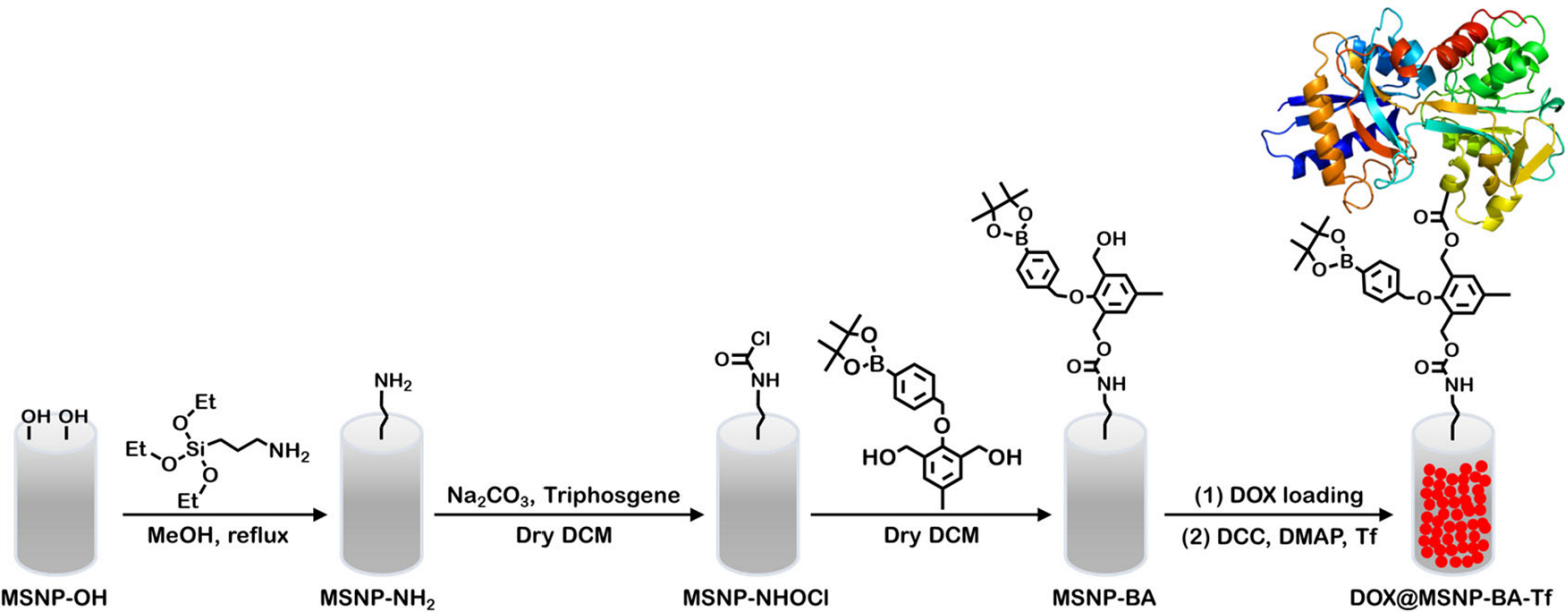
Synthesis Pathway for H_2_O_2_-Responsive Mesoporous Silica Nanoparticles (**DOX@MSNP-BA-Tf**)

Several techniques including DLS, SEM, and TEM were employed to characterize the size and shape of the MSNPs. SEM and TEM images in Figure 2a,b show that MSNP-NH_2_ has spherical and ellipsoidal shapes arranged in an ordered hexagonal pattern. Figure S1 (in the Supporting Information) indicates that the average hydrodynamic diameter of MSNP-NH_2_ is 145.3 nm. After modification with transferrin (Tf), **MSNP-BA-Tf**’s diameter increases to 232.1 nm. As shown in Figure 2c,d, **MSNP-BA-Tf** retains similar shapes. Notably, transferrin effectively seals the pores of MSNPs, preventing cargo leakage. The hexagonal arrangement was further confirmed by XRD analysis, with Figure S2 (in the Supporting Information) showing a peak at 2θ = 1.2°, allowing calculation of the interplanar lattice spacing (*d*100) of **MSNP-NH**_**2**_ as 3.95 nm using Bragg’s law. Surface area analyses presented in Figure 3 indicate that **MSNP-NH**_**2**_, **MSNP-BA**, and **RhB@MSNP-BA-Tf** have surface areas of 717.81, 162.51, and 37.69 m^2^/g, respectively. The dramatic decrease in surface area for RhB@MSNP-BA-Tf reflects RhB loading in mesopores and transferrin capping. The wall width of **MSNP-NH**_**2**_ was calculated as 1.68 nm using the difference between d100 from XRD (3.95 nm) and BJH pore size (2.27 nm), which is further supported by high resolution TEM images (Figure S3 in the Supporting Information) confirming values of *d*100, pore size, and wall width consistency.

**Figure 2:**
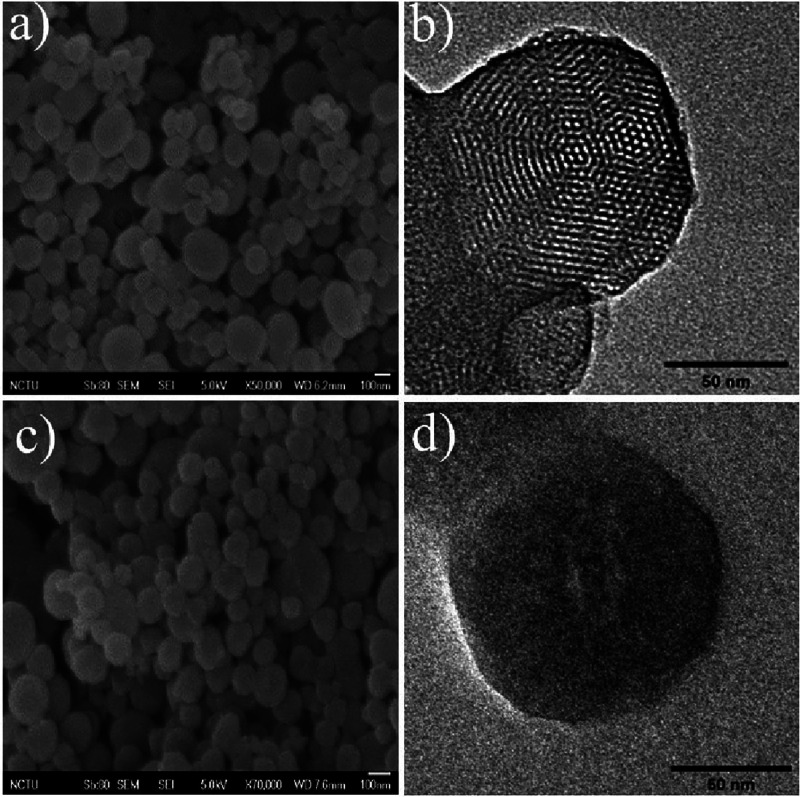
SEM and TEM images of **MSNP-NH**_**2**_ and **MSNP-BA-Tf**. Panels (a) and (b) display the SEM and TEM images of **MSNP-NH**_**2**_, respectively, while panels (c) and (d) depict the SEM and TEM images of **MSNP-BA-Tf**.

**Figure 3:**
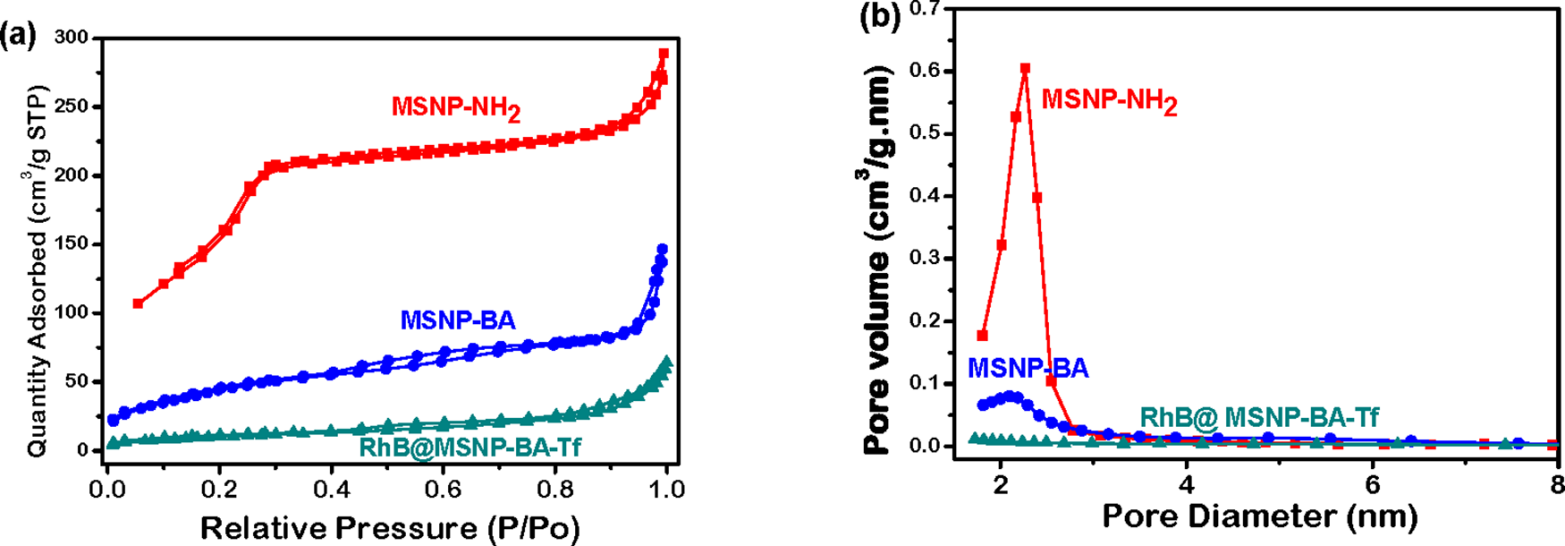
(a) Nitrogen adsorption and desorption isotherms and (b) pore size distribution of MSNP-NH_2_, MSNP-BA, and RhB@MSNP-BA-Tf.

To validate the surface modification of **MSNP-BA-Tf**, several analytical techniques were employed, including FTIR, ^13^C CP/MAS solid NMR, zeta potential, and TGA analysis. Figure S4 (in the Supporting Information) presents the FTIR spectra of various MSNPs. For MSNP–OH, the stretching vibrations of Si–OH and Si–O–Si are evident at 957 and 1048 cm^–1^, respectively. Figure S4b shows a distinct absorption peak at 1510 cm^–1^ in the spectra of **MSNP-NH**_**2**_, attributed to the NH_2_ bending mode frequency, confirming successful grafting onto MSNPs. Following modification with compound BA, Figure S4c shows the stretching of aromatic C=C bonds at 1464 cm^–1^, alongside a peak at 2962 cm-1 attributed to sp^3^ C–H stretching of compound BA, and a sharp peak at 1636 cm^–1^ from C=O stretching of the carbamate bond, confirming successful MSNP-BA modification. The attachment of Tf to MSNPs is confirmed by strong bands at 1559 and 1656 cm^–1^, characteristic of the amide skeleton of transferrin proteins, indicating successful grafting of transferrin onto MSNPs. Additionally, confirmation of MSNP-BA modification was obtained through ^13^C CP/MAS solid NMR (Figure S5 in Supporting Information), revealing resonance peaks at 9.96, 21.25, and 42.75 ppm corresponding to carbon atoms in the Si-CH_2_–CH_2_–CH_2_ moiety. The peak at 159.54 ppm confirms the presence of the C=O bond in the H_2_O_2_ trigger site of BA attached to mesoporous silica nanocarriers. Residual carbon signals observed at various ppm values in the ^13^C NMR spectrum of BA (Figure S12 in Supporting Information) further support the stability of compound BA during the grafting process.

Surface mofication was further studied by zeta potential (Figure 4a). The grafting of 3-aminopropyltriethoxysilane (APTES) onto MSNP-OH resulted in a shift in zeta potential from −26.80 mV for MSNP-OH to +36.36 mV for MSNP-NH_2_, indicating the introduction of positive charges due to the amino groups. Subsequently, the zeta potential of MSNP-BA shifted to −10.11 mV, confirming successful modification with compound BA. Given that the isoelectric point of human holo-transferrin is approximately 5.2–5.6, the negative zeta potential value of −12.74 mV for **MSNP-BA-Tf** at neutral pH reflects the presence of the negatively charged surface of transferrin, indicating successful grafting onto MSNPs. In Figure 4b, thermogravimetric analysis (TGA) profiles of various mesoporous silica nanoparticles (MSNPs) are depicted. MSNP-OH shows relatively stable weight loss between 200 to 800 °C, indicating good thermal stability. MSNP–OH, MSNP-NH_2_, MSNP-BA, and **MSNP-BA-Tf** exhibit weight losses of 10.2, 14.3, 28.9, and 45.1%, respectively. The progressive increase in weight loss reflects the decomposition of grafted organic materials, confirming successful modification of the MSNPs.

**Figure 4:**
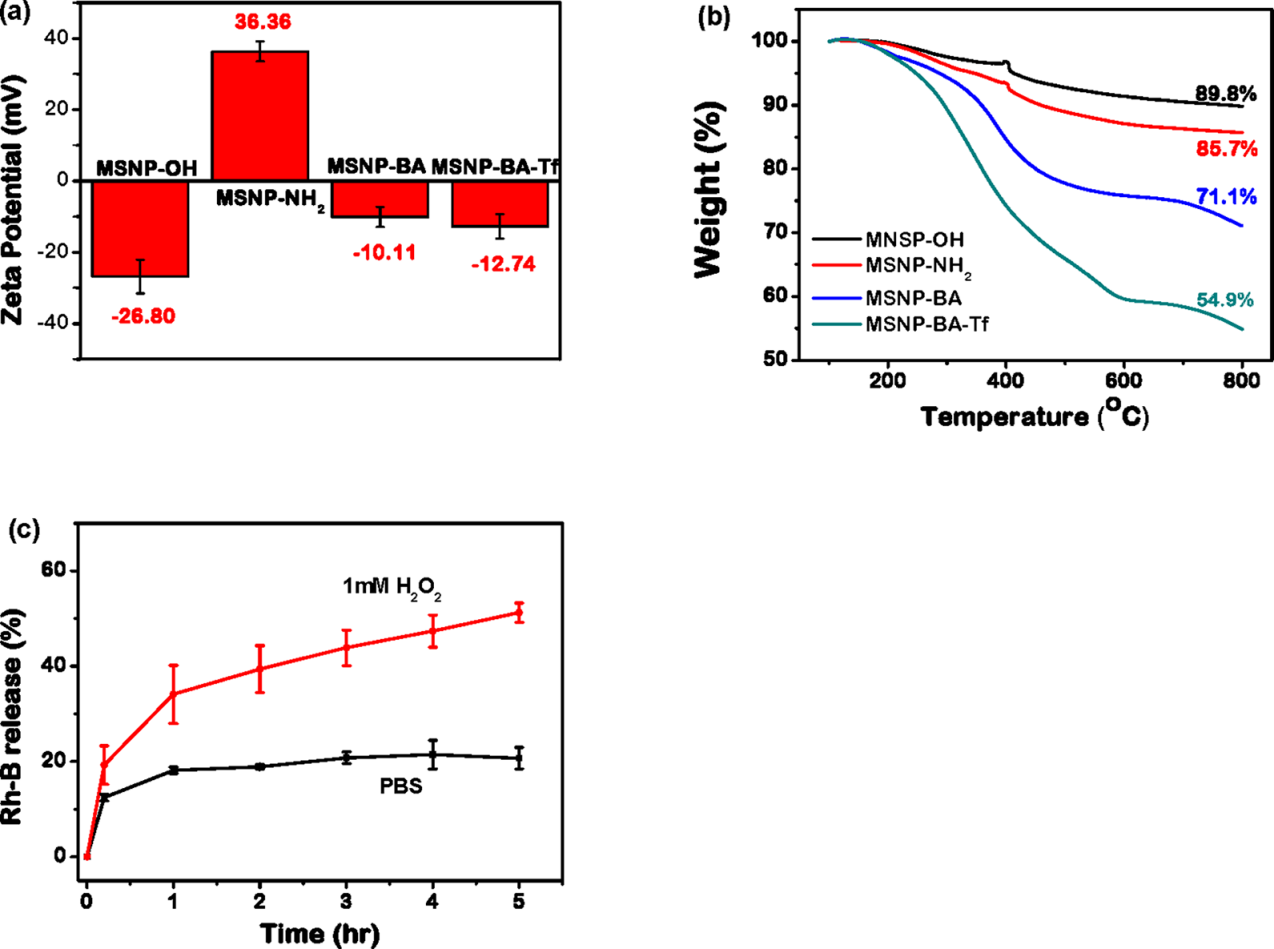
(a) Zeta potential variation and (b) thermogravimetric curves of MSNP-OH, MSNP-NH_2_, MSNP-BA, and MSNP-BA-Tf. (c) Release profiles of Rhodamine B from **MSNP-BA-Tf** loaded with RhB were studied under two conditions: PBS buffer (pH 7.4) and in the presence of 1 mM H_2_O_2_.

To determine the optimal drug loading conditions, MSNP-NH_2_ was suspended in various ratios of RhB to nanoparticle solution for 24 h. The difference in RhB absorbance before and after loading in MSNP-NH_2_ (Figure S6 in the Supporting Information) was quantified to determine the amount of RhB loaded. Figure S7 (in the Supporting Information) shows that MSNP-NH_2_ achieved a loading capacity ranging from 15 to 27 μg of RhB per milligram of MSNP-NH_2_. Notably, the best loading efficiency was observed at a solution ratio of 2:10 (RhB to nanoparticles), which minimizes drug cost and reduces waste. Following the capping of nanoparticles with transferrin, the release of cargo from RhB-loaded **MSNP-BA-Tf** was further investigated by monitoring fluorescence changes in PBS solutions containing the nanoparticles at hourly intervals. Figure 4c illustrates the suspension of RhB-loaded **MSNP-BA-Tf** in PBS buffer with or without 1 mM H_2_O_2_. In the presence of 1 mM H_2_O_2_, the release of RhB increased to 51% within 5 h. Conversely, minimal release was observed when RhB-loaded **MSNP-BA-Tf** was incubated in PBS buffer alone. Overall, transferrin-capped nanoparticles (**MSNP-BA-Tf**) effectively prevent cargo leakage, with the encapsulated drug being released promptly upon exposure to H_2_O_2_ stimulus.

To evaluate the efficacy of drug delivery to cancer cells, we employed the MTT assay to assess the cytotoxic effects of **MSNP-BA-Tf**, DOX@MSNP-BA-Tf, and free DOX on MCF-7, HCT116, and HEK293 cell lines (Figure 5). MCF-7 and HCT116 were selected due to their high transferrin receptor expression and elevated H_2_O_2_ levels, making them suitable for targeted cancer therapy. Conversely, HEK293 cells, with low transferrin receptor expression and H_2_O_2_ levels, served as normal controls. After a 24 h exposure to 120 μg/mL **MSNP-BA-Tf**, all cell types maintained over 80% viability, indicating good biocompatibility. Treatment with DOX@MSNP-BA-Tf resulted in a significant decrease in cell viability—approximately 30–45% in MCF-7 and HCT116 cells—comparable to free DOX. In contrast, HEK293 cells exhibited higher viability (77.6%) when treated with DOX@MSNP-BA-Tf compared to free DOX (38.5%). These findings suggest that DOX@MSNP-BA-Tf effectively targets tumor cells overexpressing transferrin receptors and H_2_O_2_, potentially reducing side effects in normal cells compared to free DOX. Similar results have been observed in related studies, where mesoporous silica nanoparticles (MSNs) loaded with DOX demonstrated enhanced cytotoxicity against MCF-7 cells, overcoming drug resistance mechanisms. Additionally, formulations containing chemotherapeutic compounds showed selective cytotoxicity toward cancer cells (MCF-7) while exhibiting reduced toxicity to normal cells (HEK293), highlighting the potential of targeted drug delivery systems.

**Figure 5:**
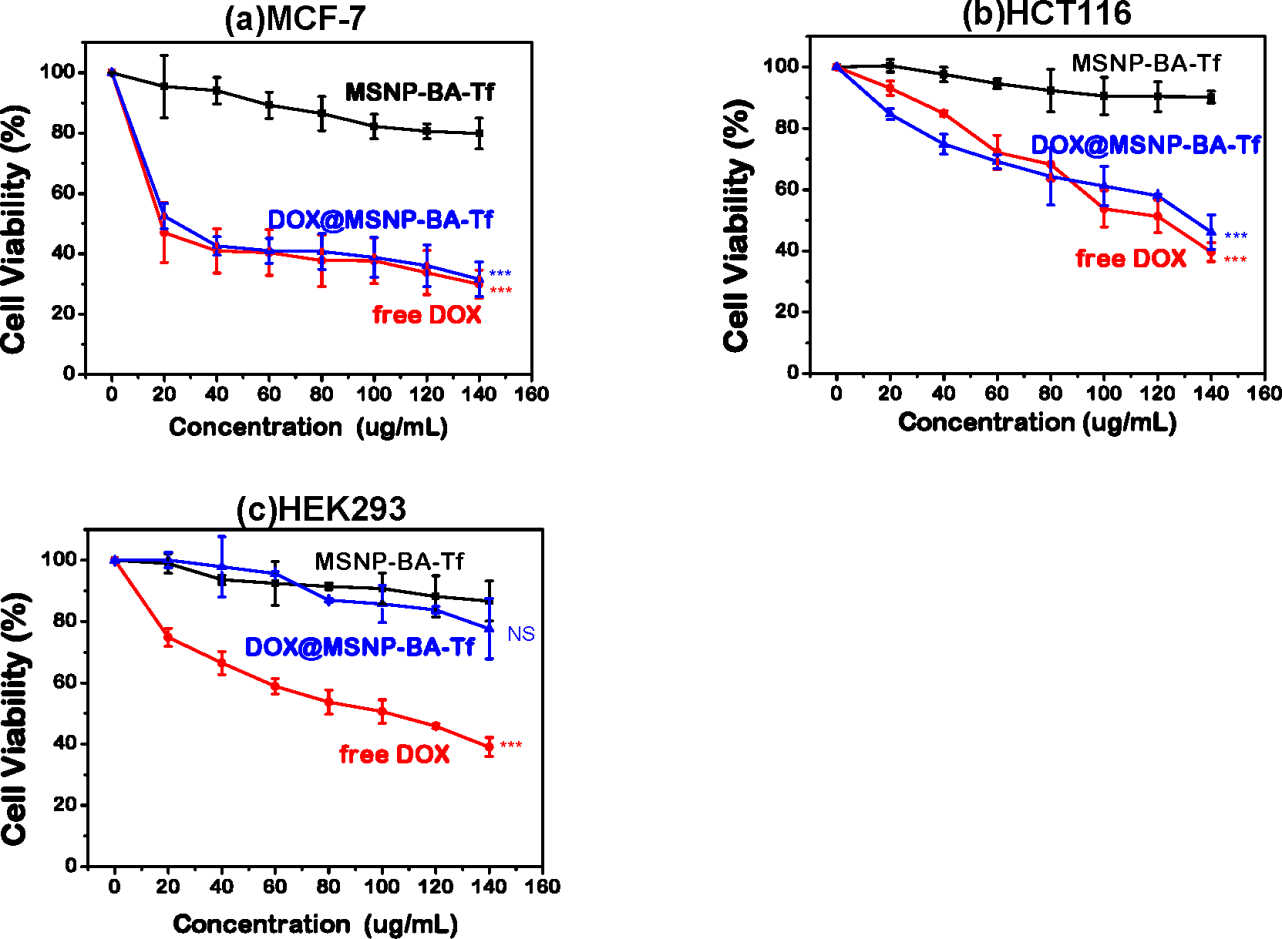
Study aimed to investigate the impact of MSNP-BA-Tf, DOX@MSNP-BA-Tf, and free DOX on the cell viability of three types of cells: (a) MCF-7, (b) HCT116, and (c) HEK293. The cells were incubated with the nanoparticles and an equivalent amount of free Dox for 24 h. The loading capacity of the MSNPs is 20 μg of DOX per milligram of MSNPs. The amount of free Dox used in the MTT assay was matched to the amount of Dox loaded in the MSNPs. *p < 0.05, **p < 0.01, ***p < 0.001, and NS denotes not significant.

The tumor-specific targeting ability of **MSNP-BA-Tf** was investigated by incubating DOX@MSNP-BA-Tf with MCF-7, HCT116, and HEK293 cells (Figure 6). MCF-7 and HCT116 represent cancer cell models, whereas HEK293 cells serve as a normal cell model. It was observed that HEK293 cells exhibited lower red fluorescence intensity compared to MCF-7 and HCT116 cells. This reduced fluorescence in HEK293 cells can be attributed to their lower expression levels of transferrin receptors and hydrogen peroxide. In contrast, MCF-7 and HCT116 cells displayed significantly higher red fluorescence, with increases of approximately 5-fold and 2.6-fold, respectively, compared to HEK293 cells. This highlights the selective targeting capability of **MSNP-BA-Tf** toward cancer cells, indicating its potential for tumor-specific drug delivery applications.

**Figure 6:**
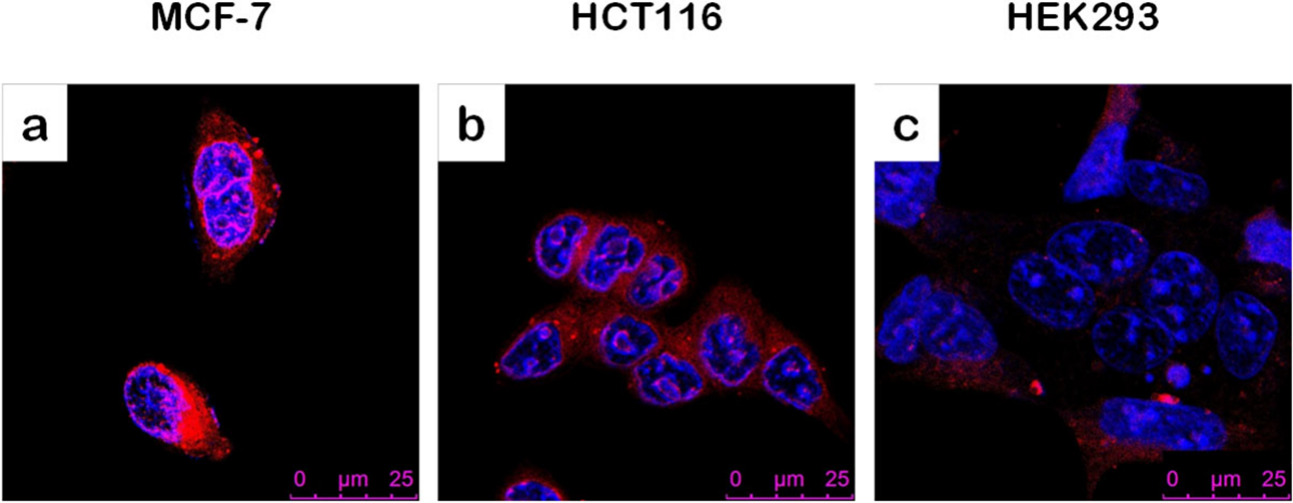
Confocal imaging of MCF-7 (a), HCT116 (b), and HEK293 (c) cells treated with DOX@MSNP-BA-Tf at a concentration of 50 μg/mL for 6 h.

Further in vitro cellular uptake was assessed by incubating DOX@MSNP-BA-Tf with MCF-7 and HCT116 cells for varying durations. Figure 7 shows a gradual increase in red fluorescence intensity within the nuclei of both cell lines over time. By 6 h, the red fluorescence fully overlapped with the blue fluorescence indicating nuclear DNA, indicating successful release of DOX from **MSNP-BA-Tf** and its interaction with DNA in the nucleus. Additionally, cellular uptake of **MSNP-BA-Tf** was evaluated using flow cytometry (Figure 8). MCF-7 and HCT116 cells were treated with RhB-free and RhB-loaded **MSNP-BA-Tf** for 4 and 6 h, respectively. Compared to control groups, **MSNP-BA-Tf** without RhB showed negligible fluorescence, indicating minimal autofluorescence from the nanoparticles. In contrast, cells treated with RhB-loaded **MSNP-BA-Tf** exhibited significantly higher RhB fluorescence compared to free RhB, suggesting efficient uptake of RhB-loaded **MSNP-BA-Tf** via transferrin receptor-mediated endocytosis. This confirms that **MSNP-BA-Tf** serves as an effective nanocarrier for targeted drug delivery in cancer therapy.

**Figure 7:**
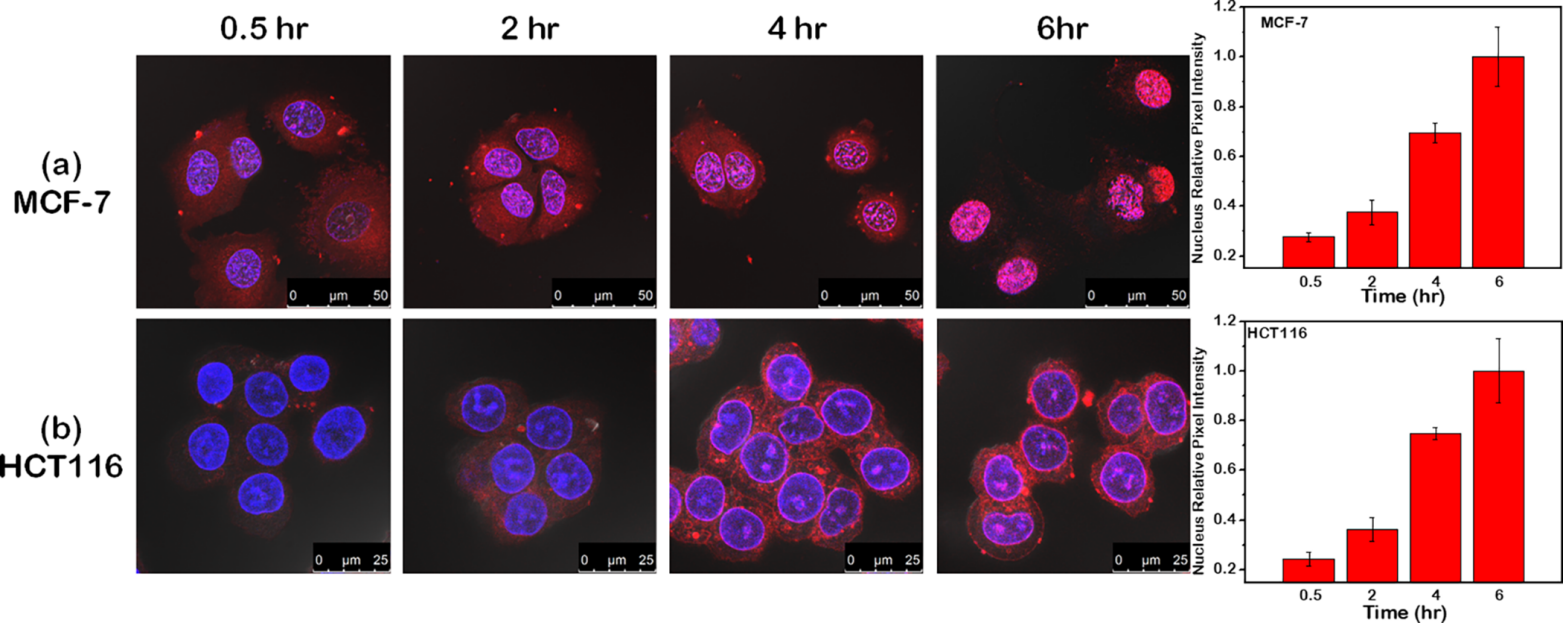
Confocal images of (a) MCF-7 and (b) HCT116 cells treated with DOX@MSNP-BA-Tf at a concentration of 100 μg/mL for 0.5, 2, 4, and 6 h. Scale bar: 50 μm. The relative pixel intensity of red fluorescence in the nucleus was quantified using ImageJ and plotted in the corresponding graphs. The red fluorescence intensity of the nucleus after 6 h was set as 1.0.

**Figure 8:**
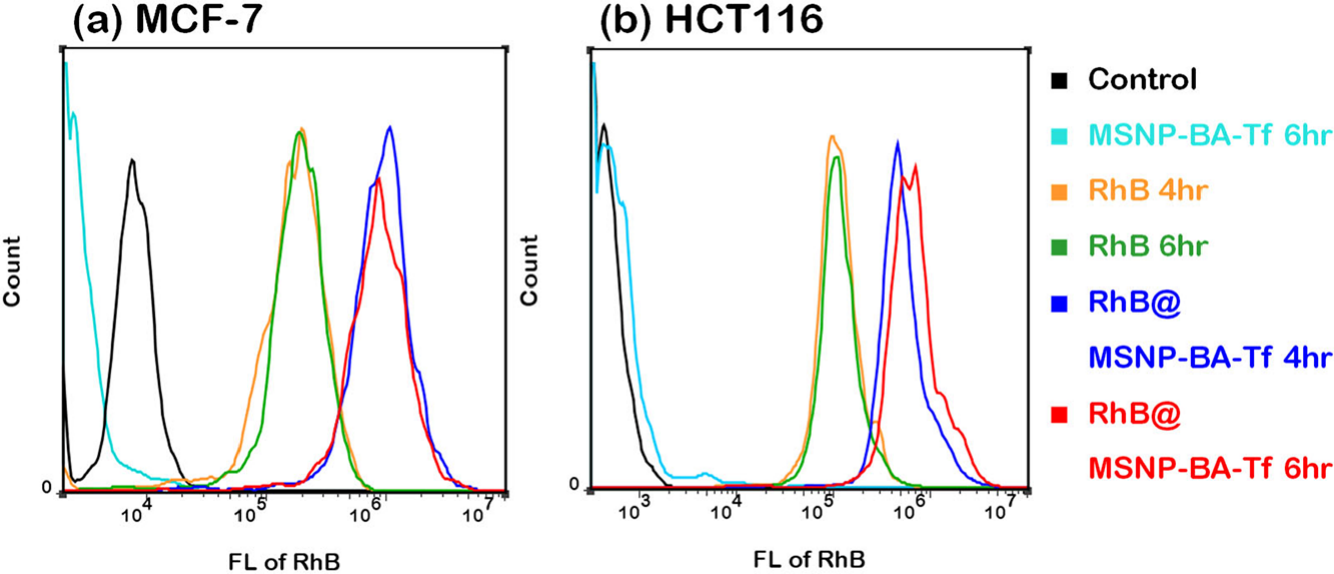
Flow cytometry analysis of (a) MCF-7 and (b) HCT116 cell lines after exposure to **MSNP-BA-Tf** (500 μg/mL), free RhB (10 μg/mL), and RhB-loaded MSNP-BA-Tf (500 μg/mL) for 4 and 6 h. The concentration of free RhB used is equivalent to that loaded in **MSNP-BA-Tf**.

To evaluate the in vivo anticancer efficacy of MSNP-BA-Tf, a colon cancer xenograft model was established to monitor tumor growth under different treatment conditions. Mice were divided into four groups and treated with PBS, free DOX, **MSNP-BA-Tf**, and DOX@MSNP-BA-Tf. Tumor size and body weight were measured daily over a 14-day period, with results shown in Figure 9. Tumor growth was similar in the PBS and **MSNP-BA-Tf** groups, indicating minimal tumor suppression with **MSNP-BA-Tf** alone. In contrast, both free DOX and DOX@MSNP-BA-Tf treatments inhibited tumor growth, with DOX@MSNP-BA-Tf demonstrating superior antitumor effects. This suggests efficient DOX accumulation within the tumor by DOX@MSNP-BA-Tf, leading to enhanced therapeutic efficacy. Importantly, no significant changes in body weight were observed in the DOX@MSNP-BA-Tf group (Figure S8 in the Supporting Information), indicating low systemic toxicity.To further assess the anticancer efficacy, hematoxylin and eosin staining of tumor tissues was performed (Figure 10). Similar to the PBS group, the **MSNP-BA-Tf** treatment showed a dense arrangement of cells. However, both free DOX and DOX@MSNP-BA-Tf treatments revealed distinct regions of apoptosis and necrosis within the tumor tissue. The presence of apoptotic and necrotic areas, even with DOX administration, confirmed effective tumor cell killing. Notably, the DOX@MSNP-BA-Tf group exhibited a larger area of damage, underscoring the enhanced antitumor efficacy of DOX@MSNP-BA-Tf. These findings highlight the ability of DOX@MSNP-BA-Tf to effectively deliver DOX to tumor sites, improving therapeutic outcomes with minimal systemic toxicity, thereby demonstrating its potential for cancer treatment applications.

**Figure 9:**
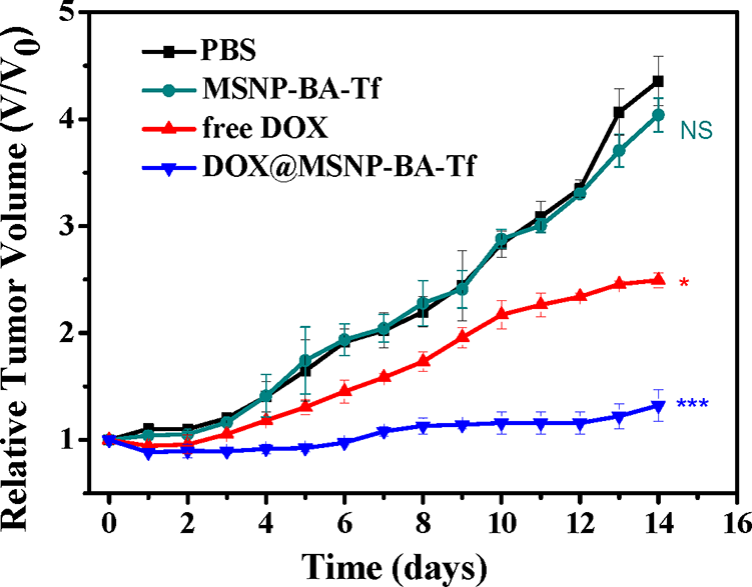
Relative tumor volume curves of HCT116 tumor-bearing mice treated with PBS (100 μL), free DOX (100 μL of 50 μg/mL), MSNP-BA-Tf (100 μL of 2.5 mg/mL), and DOX@MSNP-BA-Tf (100 μL of 2.5 mg/mL) over a period of 14 days. *p < 0.05, **p < 0.01, ***p < 0.001, and NS denotes not significant.

**Figure 10:**
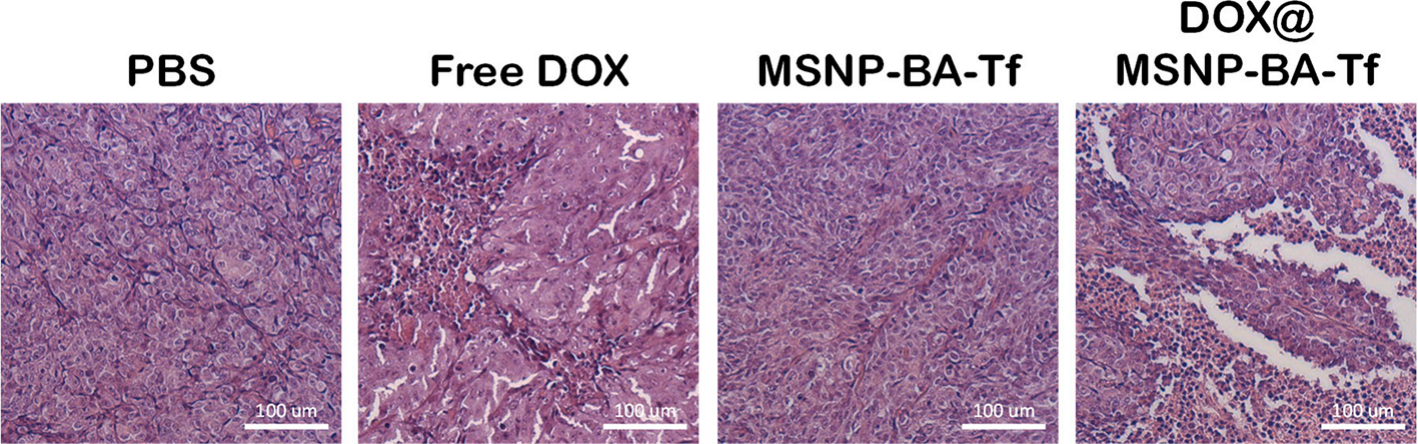
Hematoxylin and eosin staining of tumors treated with different conditions.

## Conclusions

In conclusion, our study presents a new drug delivery system employing boronic ester-functionalized mesoporous silica nanoparticles (**MSNP-BA-Tf**) for targeted cancer therapy. This system leverages the overexpression of transferrin receptors (TfR) on cancer cells and the elevated levels of hydrogen peroxide (H_2_O_2_) within tumor microenvironments. The successful synthesis and characterization of **MSNP-BA-Tf** were confirmed through comprehensive analyses, ensuring its structural integrity and functionality. We demonstrated that **MSNP-BA-Tf** effectively delivers doxorubicin (DOX), a widely used chemotherapeutic agent, specifically to cancer cells, minimizing impact on normal cells. In vitro studies, including fluorescence imaging and cytotoxicity assays, validated the selective targeting ability of **MSNP-BA-Tf**. It exhibited significant cytotoxicity against TfR-overexpressing cancer cells (MCF-7 and HCT116) while preserving the viability of normal cells (HEK293). Importantly, the responsiveness of **MSNP-BA-Tf** to H_2_O_2_ facilitated controlled drug release within cancer cells, enhancing therapeutic efficacy. Furthermore, in vivo experiments using a colon cancer xenograft model demonstrated that DOX@MSNP-BA-Tf effectively inhibited tumor growth, showing superior efficacy compared to free DOX and MSNP-BA-Tf alone. The negligible changes in body weight observed in the DOX@MSNP-BA-Tf group indicated minimal systemic toxicity, highlighting its safety profile. Overall, our findings underscore the potential of **MSNP-BA-Tf** as a promising nanocarrier for cancer-specific drug delivery, utilizing an active targeting approach.

## Supplementary Material



## Notes

^1^H NMR, ^13^C NMR, and mass spectra for all compounds, additional experimental details, materials, and methods (PDF)
