# Supplementary material for: H2O2‑Responsive Boronic Ester-Modified Mesoporous Silica Nanocarrier for TfR Mediated Tumor-Specific Drug Delivery Applications
Source: ACS Appl Bio Mater. 2025 Jun 23;8(7):6079–87. doi: 10.1021/acsabm.5c00645 (PMC12284862; doi:10.1021/acsabm.5c00645)
Supplement: Supplementary file 1 [file mt5c00645_si_001.pdf]

Supporting Information For,

H_2O_2-Responsive Boronic Ester-Modified Mesoporous Silica Nanocarrier for TfR Mediated tumor-specific Drug Delivery applications

Hsiao-Yen Lee[1], Natesan Thirumalaivasan[2] and Shu-Pao Wu[1,*]

[1]Department of Applied Chemistry, National Yang Ming Chiao Tung University, Hsinchu, 30010, Taiwan, Republic of China

[2]Department of Periodontics, Saveetha Dental College and Hospitals, Saveeta Institute of Medical and Technical Sciences (SIMATS), Chennai 600077, India

*To whom correspondence should be addressed:

Tel.: +886-3-5712121-ext 56506; Fax: +886-3-5723764; email:spwu@nycu.edu.tw

Contents

Materials and Chemicals

Instruments

Drug Loading Efficiency Assessment Using Rhodamine B in $MSNP-NH_2$

Scheme S1. Synthesis procedure of BA

Scheme S2 Mechanism of the arylboronic ester group undergoes oxidation degradation upon exposure to H_2O_2

Synthesis of Compound 1

Synthesis of Compound 2

Synthesis of BA

Figure S1. Size distribution of (a) $MSNP-NH_2$ and (b) MSNP-BA-Tf by DLS.

Figure S2. Powder X-ray diffraction (XRD) pattern showing the low-angle and interplanar lattice spacing of **$MSNP-NH_2$**.

Figure S3. The enlarged HR-TEM image of $MSNP-NH_2$ and its measurements of d_{100}, pore size and wall width which were obtained by Image J.

Figure S4. The FT-IR analysis was performed on MSNP-OH, $MSNP-NH_2$, MSNP-BA, and **MSNP-BA-Tf**.

Figure S5. ^{13}C CP/MAS solid NMR analysis of MSNP-BA.

Figure S6. Absorbance of rhodamine B before and after loading in $MSNP-NH_2$ using various weight ratios of rhodamine B to $MSNP-NH_2$ in 1 mL PBS

Figure S7. Loading efficacy of rhodamine B in $MSNP-NH_2$ in different ratios of rhodamine B to $MSNP-NH_2$

Figure S8. Relative body weight curves of HCT116 tumor-bearing mice treated with PBS (100 μL), free DOX (100 μL of 50 μg/mL), MSNP-BA-Tf (100 μL of 2.5 mg/mL), and DOX-loaded MSNP-BA-Tf (100 μL of 2.5 mg/mL) over a period of 14 days.

Figure S9. Confocal imaging of HCT116 cells treated with free DOX at a concentration of 125 μg/mL for two hours.

Figure S10. Confocal imaging of HCT116 cells treated with DOX@MSNP-BA at a concentration of 50 μg/mL for two hours.

Figure S11. ^{1}H NMR (400 MHz) spectrum of BA in $CDCl_3$.

Figure S12. ^{13}C NMR (100 MHz) spectrum of BA in $CDCl_3$

Figure S13. HR-FD MS spectrum of BA.

Materials and Chemicals

Tetraethylorthosilicate (TEOS), magnesium sulfate (MgSO4), and sodium carbonate (Na_2CO_3) were purchased from Showa. Dimethylformamide was sourced from Echo Chemical Co., Ltd., and methanol from Aencore. Triphosgene, 3-(4,5-dimethylthiazol-2-yl)-2,5-diphenyltetrazolium bromide (MTT), N,N'-dicyclohexylcarbodiimide (DCC), Cetyltrimethylammonium bromide (CTAB), 4-dimethylaminopyridine (DMAP), 2,6-bis-(hydroxymethyl)-p-cresol, imidazole, tert-butyldimethylsilyl chloride, rhodamine B (RhB), and p-toluenesulfonic acid monohydrate were procured from Alfa Aesar and TCI for the study. Additionally, 3-aminopropyltriethoxysilane (APTS), doxorubicin (DOX), transferrin (Tf), and hydrogen peroxide (H_2O_2) were sourced from Sigma-Aldrich.

Instruments

A dual approach was employed to visualize the nanocarriers, utilizing transmission electron microscopy (TEM; JEOL JSM-7401F) and scanning electron microscopy (SEM; JEOL JEM-2100). The electrokinetic potential and size distributions of the mesoporous silica nanoparticles (MSNPs) were analyzed using the Otsuka ELSZ-2000 instrument. X-ray diffraction (XRD) patterns of the nanoparticles were measured at SWAXS from NSRRC BL23A. Nanoparticle characterization included collecting N2 adsorption-desorption isotherms, surface area, and pore size data using the Micromeritics Tristar 3000 equipment. FT-IR spectra of the MSNPs were obtained using the PerkinElmer Spectrum One, and 13C solid-state nuclear magnetic resonance spectra were analyzed with the Bruker Avance III 400. UV/visible spectroscopic analysis was conducted using an Agilent 8453 UV/visible spectrophotometer. Fluorescence spectra were measured with a HITACHI F-7000 fluorescence spectrophotometer. Absorbance in the MTT assay was measured using a microplate spectrophotometer (Thermo Scientific Multiskan GO). Optical laser scanning microscope images were acquired with the Leica TCS-SP5-X AOBS. Nuclear magnetic resonance spectra were obtained using an Agilent 400 NMR spectrometer.

Drug Loading Efficiency Assessment Using Rhodamine B in MSNP-NH_2

Drug loading efficiency was used to study drug loading in nanoparticles. Here, we used RhB to replace the DOX for drug loading test. Different amounts of rhodamine B (0.5, 1, 2,3,4 mg/mL) were prepared and

MSNP-NH$_2$ (10 mg) were incubated with the rhodamine B solution. By measuring the absorption change, the concentration difference can be calculated by Beer's law. The profile of drug loading efficiency with different concentration of rhodamine B is obtained. The loading capacity of **MSNP-NH$_2$** was 15-27 μg RhB per milligram **MSNP-NH$_2$**. When **MSNP-NH$_2$** (10 mg) were incubated with 2 mg/mL RhB, **MSNP-NH$_2$** had the best loading efficacy which can have the lowest drug cost and prevent waste of the drug. **MSNP-NH$_2$** (1 mg) can hold RhB (20 μg). In addition, DOX also do the loading experiment. **MSNP-NH$_2$** (1 mg) can hold DOX (20 μg), which was almost the same as RhB result indicating the validity of the replacement of DOX to RhB.

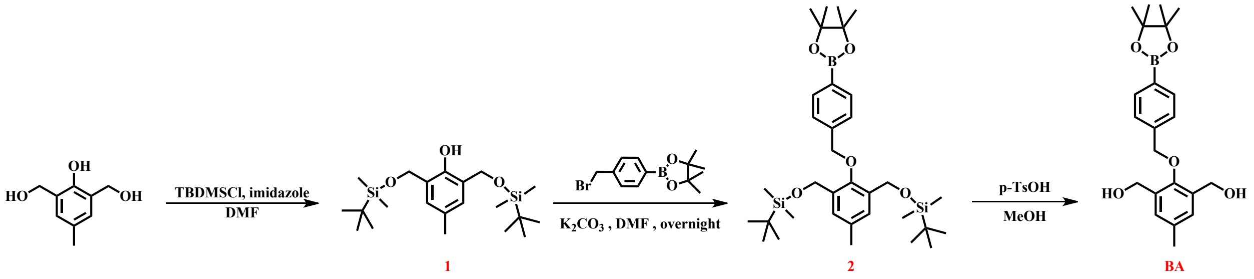

Scheme S1. Synthesis procedure of BA.

Synthesis of Compound 1

2,6-bis-(Hydroxymethyl)-p-cresol (1 g, 5.94 mmol) and imidazole (890 mg, 13.07 mmol) were dissolved in dry DMF (10 mL) and cooled in an ice bath to 0 °C. Subsequently, a solution of TBDMSCl (1.97 g, 13.08 mmol) in dry DMF was added dropwise into the solution. Then the reaction was stirred at RT for 2 hours and monitored by TLC. After the reaction was completed, the mixture was extracted with EA and brine. The organic layer was dried over $MgSO_4$ and evaporated under reduced pressure to give compound **1** as a yellow oil, yield: 85%. ^{1}H NMR (400 MHz, $CDCl_3$) δ: 8.04 (s, 1H), 6.92 (s, 2H), 4.84 (s, 4H), 2.27 (s, 3H), 0.95 (s, 18H), 0.14 (s, 12H).

Synthesis of Compound 2

A solution of compound **1** (796 mg, 2 mmol) in dry DMF (5 mL) was stirred and cooled to 0 °C. K_2CO_3 (332 mg, 2.4 mmol) was added and the mixture was stirred for 10 minutes at 0 °C. Subsequently, 4-bromomethylphenylboronic acid pinacol ester (625 mg, 2 mmol) was further added and stirred at RT overnight. The reaction was monitored by TLC. After the reaction was completed, the mixture was extracted with EA and brine, and then the organic layer was dried over $MgSO_4$. After the removal of the solvent, compound **2** was obtained as light yellow oil, yield:

65%. ^{1}H NMR (400 MHz, $CDCl_3$) δ: 7.84 (d, 2H, *J* = 7.6 Hz), 7.42 (d, 2H, *J* = 7.6 Hz), 7.17 (s, 2H), 4.89 (s, 2H), 4.70 (s, 4H), 2.34 (s, 3H), 1.35 (s, 12H), 0.91 (s, 18H), 0.07 (s, 12H).

Synthesis of BA

To a solution of compound **2** (305 mg, 0.5 mmol) in MeOH (3 mL), little amount of *p*-TsOH (18.3 mg, 0.09 mmol) was added. The mixture was stirred at RT and monitored by TLC. After the reaction was completed, the solvent was removed under reduced pressure. The residue was dissolved in EA and washed with brines. The organic layer was dried over $MgSO_4$ and filtered. After the removal of the solvent, BA was obtained as a light yellow oil, yield: 61%. ^{1}H NMR (400 MHz, $CDCl_3$) δ: 7.83 (d, 2H, *J* = 7.6 Hz), 7.42 (d, 2H, *J* = 7.6 Hz), 7.15 (s, 2H), 4.94 (s, 2H), 4.65 (s, 4H), 2.32 (s, 3H), 1.35 (s, 12H). ^{13}C NMR (100 MHz, $CDCl_3$) δ: 152.76, 140.00, 135.28, 134.61, 133.99, 129.78, 127.33, 84.05, 76.97, 61.20, 25.01, 20.98. HR-FD Mass [M]: calcd. for $C_{22}H_{29}BO_5$: 384.2108; found 384.2106.

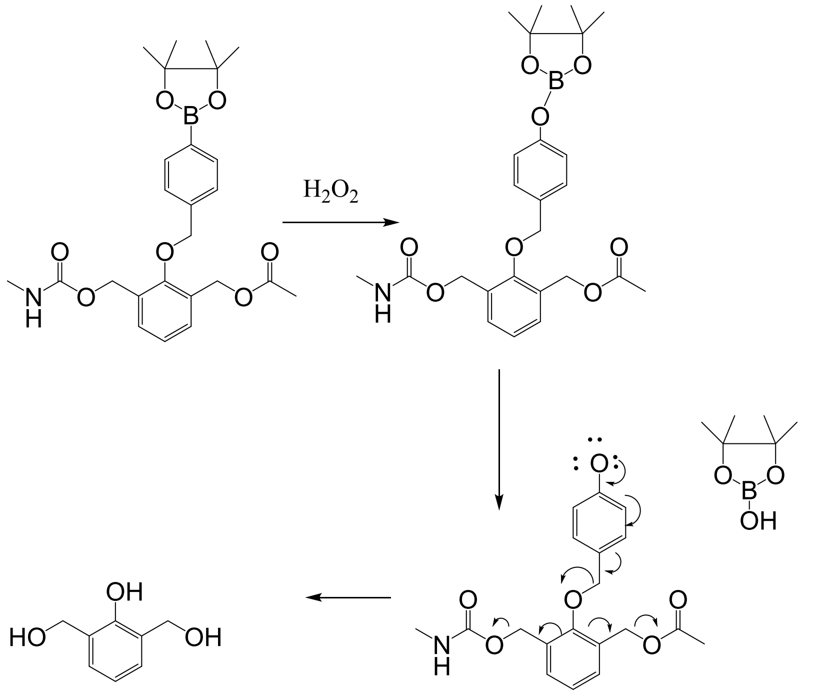

Scheme S2 Mechanism of the arylboronic ester group undergoes oxidation degradation upon exposure to H_2O_2

(a)

(b)

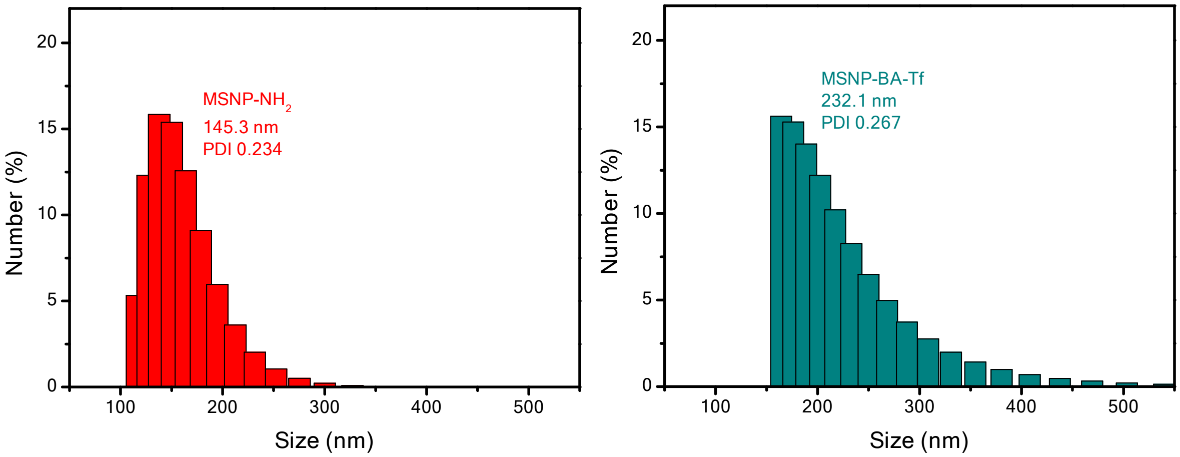

Figure S1. Size distribution of (a) MSNP-NH_2 and (b) MSNP-BA-Tf by DLS.

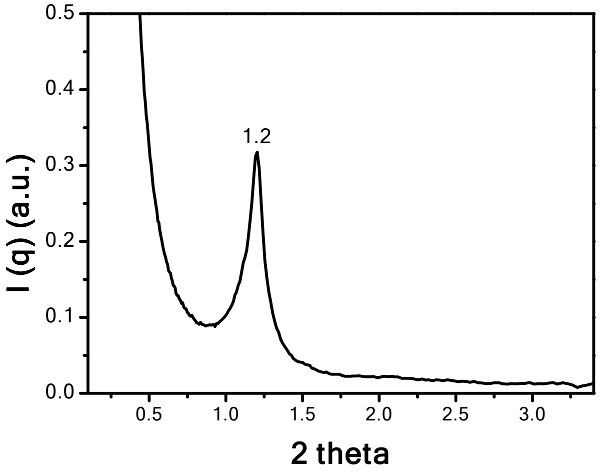

Bragg's Law

$$n\lambda = 2\,\mathrm{d}\sin\theta$$

(n = 1, λ = 0.826567 Å)

$$d = \frac{n\lambda}{2\sin\theta}$$

$$d = \frac{1 * 0.826567}{2 * \sin(\frac{1.2}{2})} = 39.47\ \text{Å} = 3.947\ nm$$

Figure S2. Powder X-ray diffraction (XRD) pattern showing the low-angle and interplanar lattice spacing of **MSNP-NH_2**.

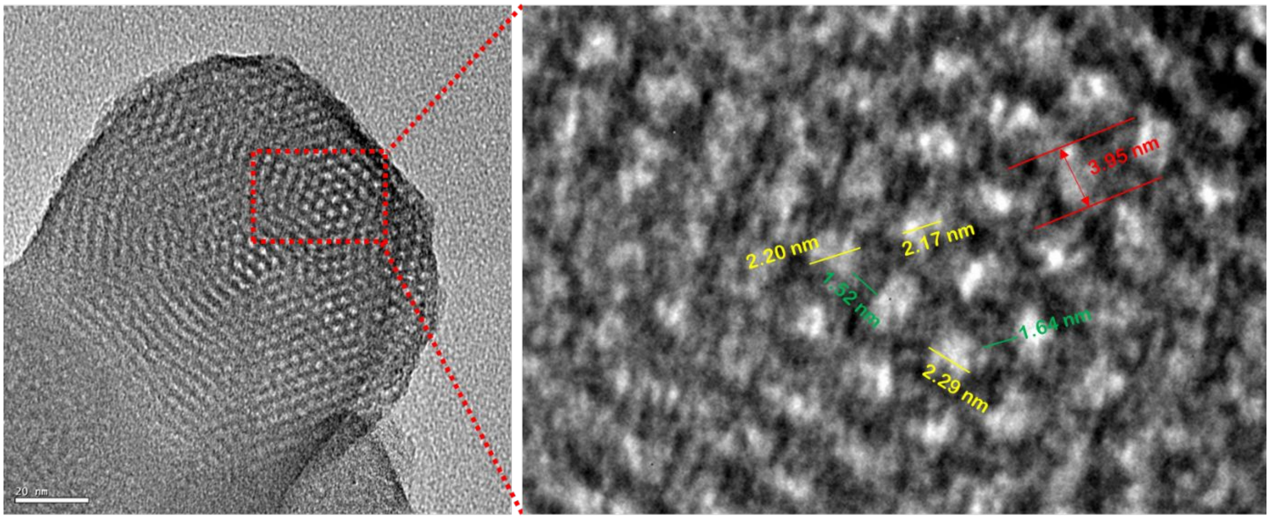

Figure S3. The enlarged HR-TEM image of MSNP-NH_2 and its measurements of d_{100}, pore size and wall width which were obtained by Image J.

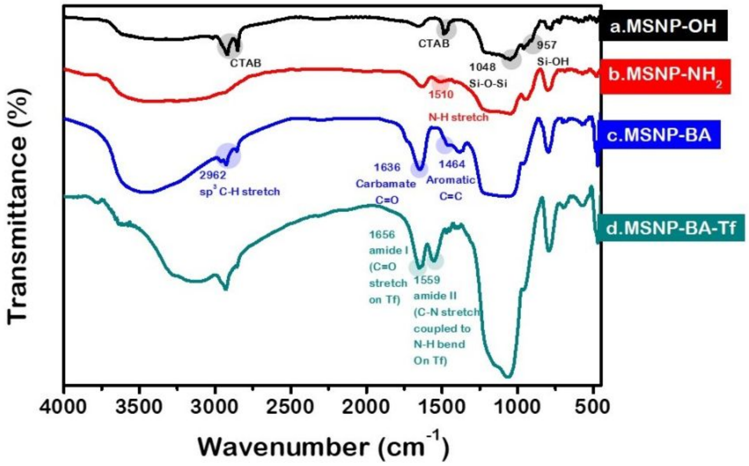

Figure S4. The FT-IR analysis was performed on MSNP-OH, MSNP-NH_2, MSNP-BA, and **MSNP-BA-Tf**.

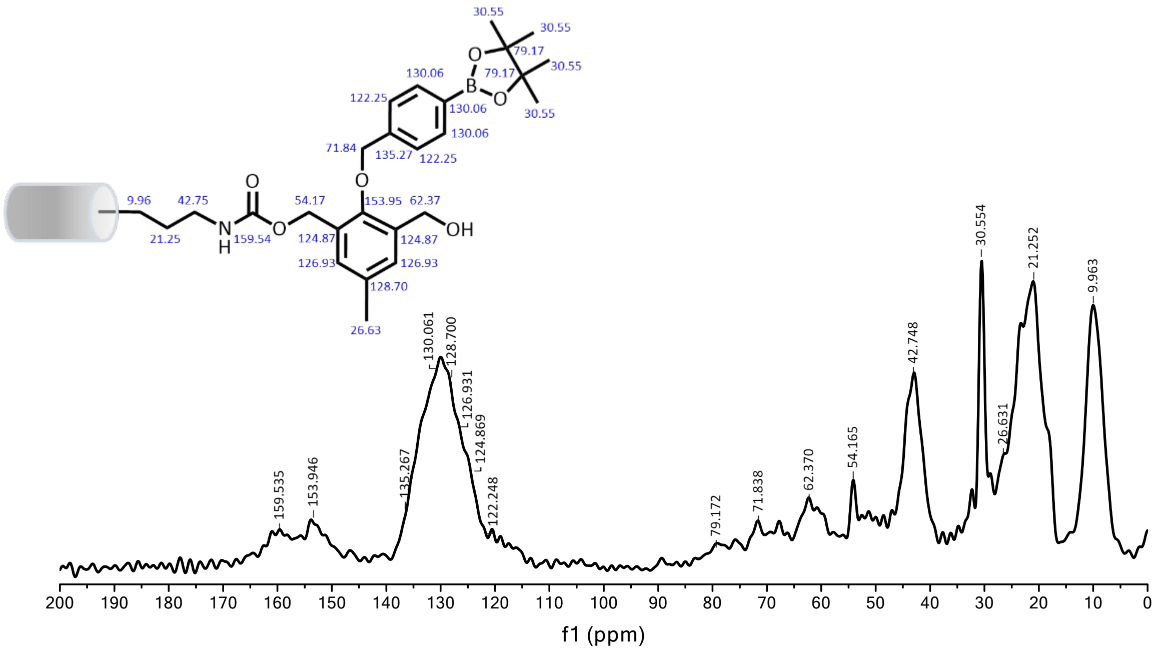

Figure S5. ^{13}C CP/MAS solid NMR analysis of MSNP-BA.

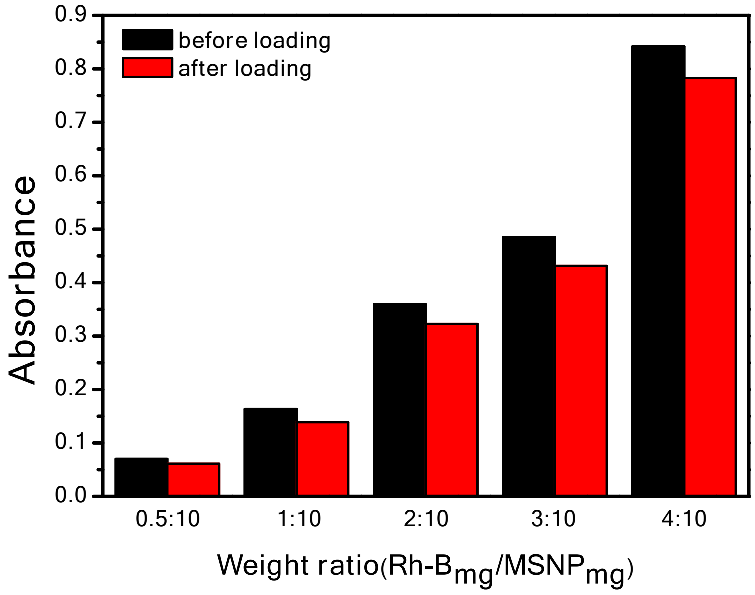

Figure S6. Absorbance of rhodamine B before and after loading in MSNP-NH_2 using various weight ratios of rhodamine B to MSNP-NH_2 in 1 mL PBS.

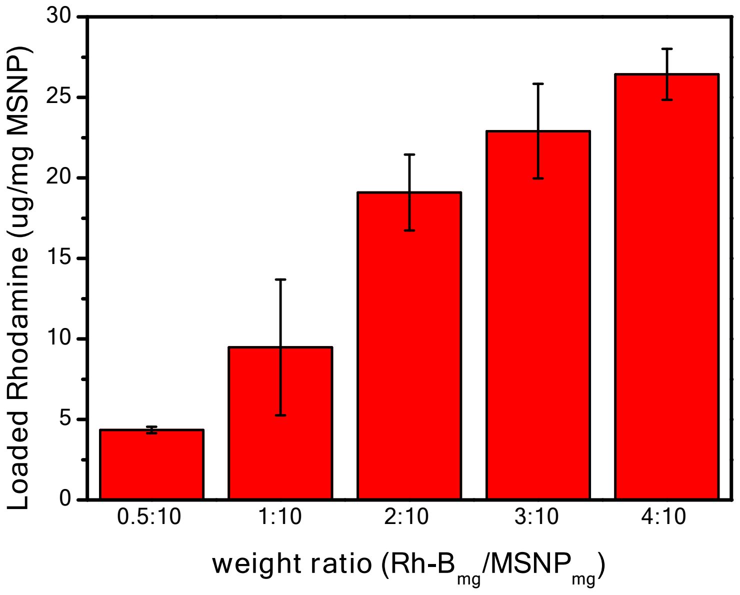

Figure S7. Loading efficacy of rhodamine B in MSNP-NH_2 in different ratios of rhodamine B to MSNP-NH_2.

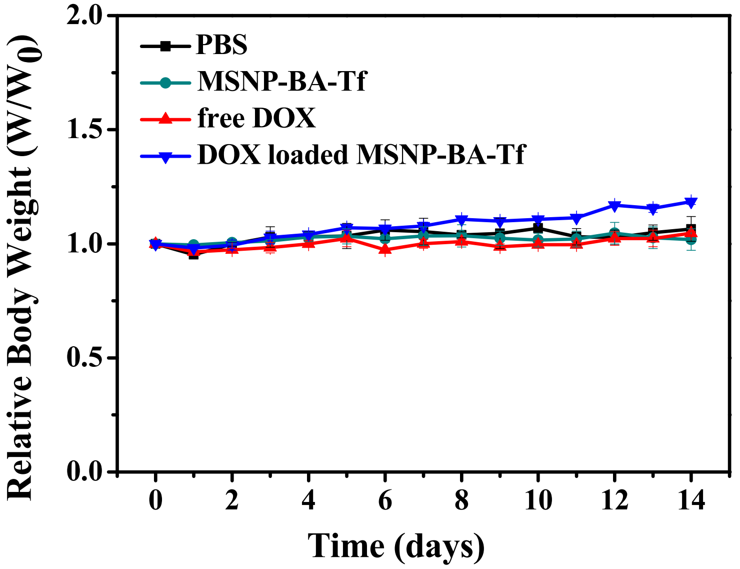

Figure S8. Relative body weight curves of HCT116 tumor-bearing mice treated with PBS (100 μL), free DOX (100 μL of 50 μg/mL), MSNP-BA-Tf (100 μL of 2.5 mg/mL), and DOX-loaded MSNP-BA-Tf (100 μL of 2.5 mg/mL) over a period of 14 days.

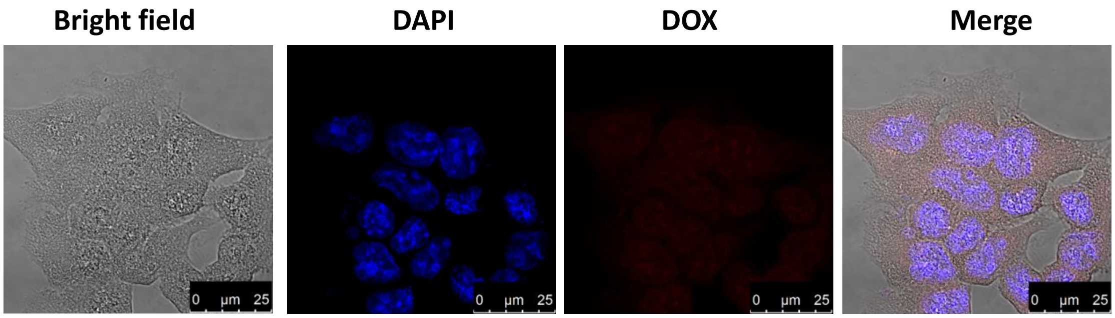

Figure S9. Confocal imaging of HCT116 cells treated with free DOX at a concentration of 125 μg/mL for two hours.

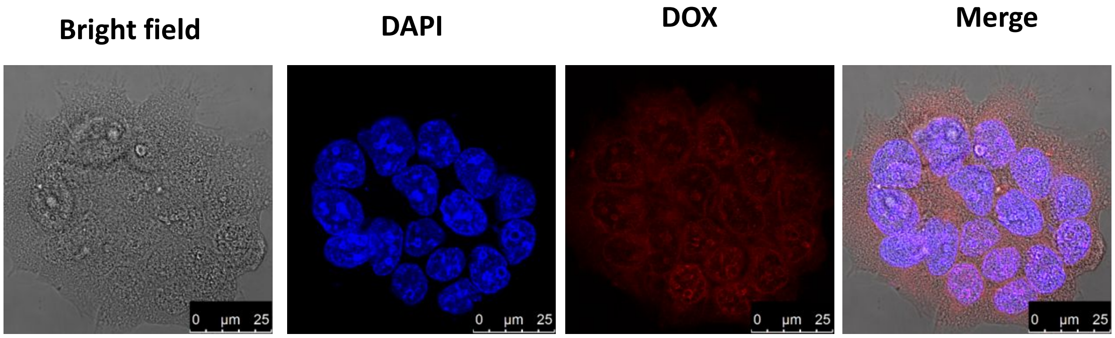

Figure S10. Confocal imaging of HCT116 cells treated with DOX@MSNP-BA at a concentration of 50 μg/mL for two hours.

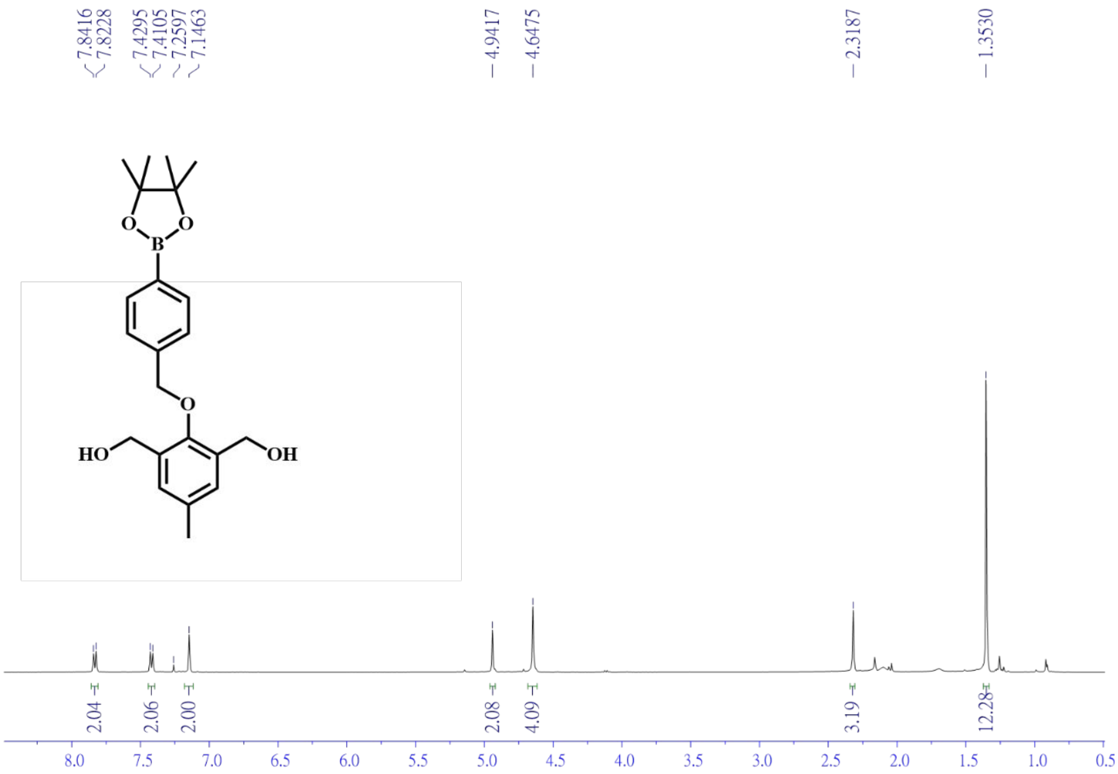

Figure S11. ^{1}H NMR (400 MHz) spectrum of BA in $CDCl_3$.

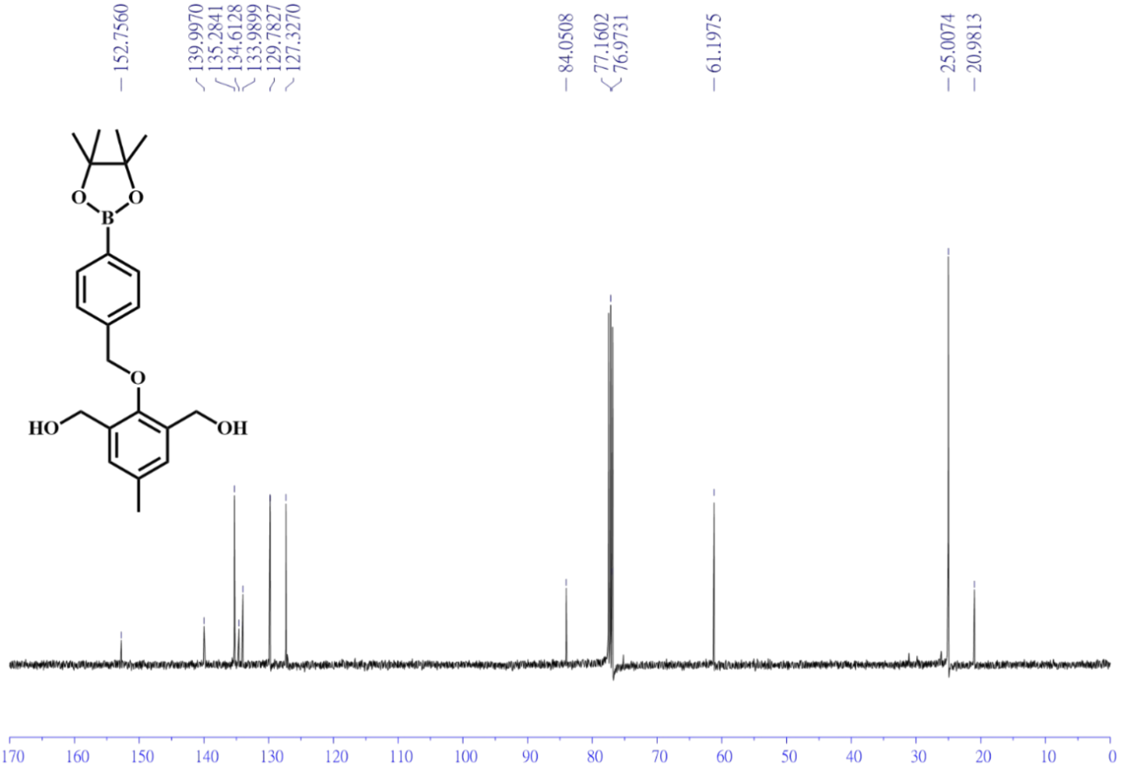

Figure S12. ^{13}C NMR (100 MHz) spectrum of BA in $CDCl_3$.

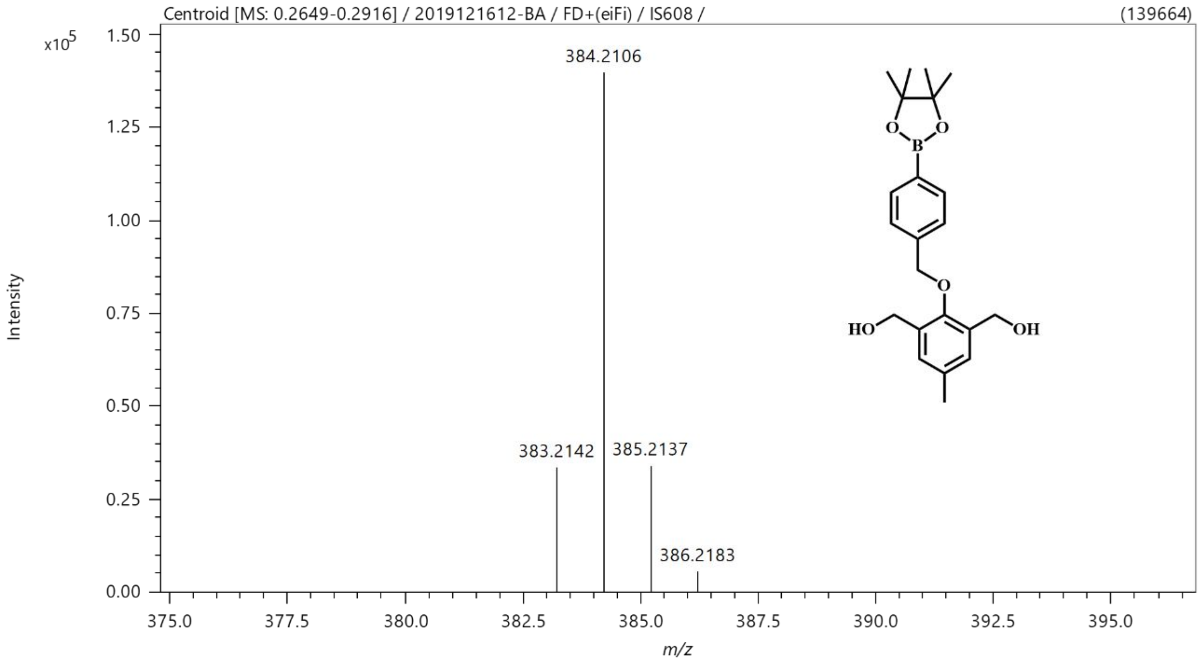

Figure S13. HR-FD MS spectrum of BA.
